# Probing the Protein-Protein Interaction between the ATRX_ADD_ Domain and the Histone H3 Tail

**DOI:** 10.3390/molecules25071500

**Published:** 2020-03-25

**Authors:** Angela M. Zaino, Radha Charan Dash, M. Kyle Hadden

**Affiliations:** Department of Pharmaceutical Sciences, University of Connecticut, 69 North Eagleville Rd, Storrs, CT 06029-3092, USA; angela.zaino@uconn.edu (A.M.Z.); radha.dash@uconn.edu (R.C.D.)

**Keywords:** protein-protein interactions, cancer, isothermal titration calorimetry, virtual screening, fluorescence polarization, AlphaScreen

## Abstract

While loss-of-function mutations in the *ATRX* gene have been implicated as a driving force for a variety of pediatric brain tumors, as well as pancreatic neuroendocrine tumors, the role of ATRX in gene regulation and oncogenic development is not well-characterized. The ADD domain of ATRX (ATRX_ADD_) localizes the protein to chromatin by specifically binding to the histone H3 tail. This domain is also a primary region that is mutated in these cancers. The overall goal of our studies was to utilize a variety of techniques (experimental and computational) to probe the H3:ATRX_ADD_ protein-protein interaction (PPI). We developed two biochemical assays that can be utilized to study the interaction. These assays were utilized to experimentally validate and expand upon our previous computational results. We demonstrated that the three anchor points in the H3 tail (A1, K4, and K9) are all essential for high affinity binding and that disruption of more than one contact region will be required to develop a small molecule that disrupts the PPI. Our approach in this study could be applied to other domains of ATRX, as well as PPIs between other distinct proteins.

## 1. Introduction

The *ATRX* gene was discovered in 1995, while studying a unique type of mental retardation that was paired with alpha thalassemia [1]. This syndrome, Alpha-Thalassemia mental-retardation X-linked, was linked to a mutation in XH2 (X-linked Helicase II) [1]. XH2 was subsequently renamed ATRX, because of its causative role in the development of ATRX syndrome. ATRX is a member of the SWI/SNF family of chromatin remodelers, which recognize specific histone modifications and utilize ATP-dependent processes to regulate gene expression. ATRX mediates this function through three primary domains: (1) the N-terminal ATRX-DNMT3-DNMT3L domain (ATRX_ADD_), (2) a centrally-located DAXX-binding motif (death-associated protein 6, ATRX_DBM_), and (3) the C-terminal SNF2 helicase-like, ATPase domain (ATRX_ATP_) [2,3]. The general mechanism through which ATRX regulates gene expression is in coordination with DAXX, which is a chaperone for the replication-independent deposition of histone variant H3.3 into the nucleosome. Histone variant H3.3 was originally thought to be associated with transcriptionally active regions of DNA; however, the ATRX/DAXX complex results in H3.3 accumulation in centromeres and telomeres, which are transcriptionally silent [4]. 

The mutations in ATRX that cause ATRX syndrome are located in either the ATRX_ADD_ or the ATRX_ATP_ domain [5]. Recent cancer genome studies have identified loss-of-function mutations in ATRX_ADD_ in multiple pediatric and adult cancers [6,7,8,9,10]. The increased prevalence of ATRX loss-of-function in ATRX syndrome and cancer suggests that it plays an essential role in regards to proper chromatin structure and/or gene regulation in these tissues; however, our overall understanding of both the normal and oncogenic roles of ATRX is still at a preliminary stage. The primary role of ATRX_ADD_ in ATRX function is to localize the protein to the nucleosome through a protein-protein interaction (PPI), with the histone H3 tail. ATRX_ADD_ is a methyl-lysine binding domain that has a unique affinity for histone H3, which is strongest when lysine 9 is trimethylated and lysine 4 is unmodified (H3K9me3/K4me0) [11,12]. Interestingly, the H3K9me3 binding pocket in ATRX_ADD_ is structurally atypical compared to classical methyl-lysine binding domains. The majority of other methyl-lysine binding domains identified to date are characterized by an ‘aromatic cage’, in which Phe, Tyr, and Trp residues are instrumental in binding the modified lysine residue [13]. By contrast, the H3K9me3 binding site on ATRX_ADD_ is rich in polar residues and key intermolecular binding interactions, including a series of nonconventional carbon-oxygen hydrogen bonds between the ε-N-trimethyl lysine moiety and multiple binding site residues [11,12,14].

We recently performed a series of computational studies to probe the key intermolecular interactions between H3 and ATRX_ADD_, as a first step to gaining a better understanding of ATRX function [15]. Our goal with these follow-up studies was two-fold. First, we sought to identify small molecules capable of disrupting the H3K9me3:ATRX_ADD_ PPI to use as chemical probes, to more clearly define the epigenetic and cellular mechanisms through which the ATRX_ADD_ regulates proper gene expression. To accomplish this first goal, we developed primary and secondary assays that were subsequently utilized in biochemical and virtual screening approaches to explore the small molecule regulation of the PPI. Our second goal was to more fully validate our computational findings in an experimental setting, by determining whether truncated or modified H3 peptides could displace the H3K9me3:ATRX_ADD_ PPI. Herein, we report the development and optimization of two orthogonal biochemical assays and their application toward probing the H3K9me3:ATRX_ADD_ PPI. 

## 2. Results

### 2.1. Isothermal Titration Calorimetry Studies

Our initial step in the development of both assays was to utilize isothermal titration calorimetry (ITC) to identify suitable reagents (H3_1–15_ peptides and ATRX_ADD_ domain proteins), to ensure robust and reproducible protocols. We utilized ITC to replicate the binding studies previously performed, as well as to determine whether labelling the H3 peptide would affect its binding to the ATRX_ADD_ [11]. For the unlabeled H3 peptides, we observed the same trend previously demonstrated, i.e. that sequential methylation at the K9 position enhanced the binding affinity between the first fifteen residues of the H3 tail (H3_1–15_) and ATRX_ADD_ (Table 1). Interestingly, our K_d_ values were approximately 30–100 fold higher than those previously determined by ITC for H3_1–15_ peptide binding to ATRX_ADD_. There are many possible reasons for this discrepancy, including differences in buffers utilized across the ITC studies or potential impurities in our peptide or ATRX_ADD_ samples that affected overall binding affinity. In an attempt to address these discrepancies while also exploring a more rapid method to determine binding affinities for H3_1–15_ and ATRX_ADD_, we determined K_d_ values for these four peptides through microscale thermophoresis (MST). Through this method, the binding affinity for the unlabeled and mono-methylated H3_1–15_ peptides were lower than our ITC-derived values and correlated well with the affinity previously determined by ITC (Table 1, Supplementary Figure S1). In addition, while the H3_1–15_K9me3 peptide demonstrated the highest affinity for ATRX_ADD_ compared to the other peptides, the established binding affinity trend from non- to tri-methylated H3_1–15_K9 was not realized through the MST protocol. 

It is important to note that a previous fluorescence-titration assay determined K_d_ values for binding of a small set of H3 peptides to ATRX_ADD_ [14]. The K_d_ values reported for H3K9me0 (>80 µM) and H3K9me3 (1.3 µM) are comparable to the IC_50_ values we obtained in our FP assay (see below). Taken together, these results clearly indicate that the absolute K_d_ value can vary across experimental procedures and that the absolute value is less important than the binding/activity trend. Since our primary goal for these initial binding studies was to replicate the previously identified binding trend across the various K9 methylated H3_1–15_ peptides, we continued with the ITC studies, without further optimization of our protocols or reagents.

**Figure 1:**
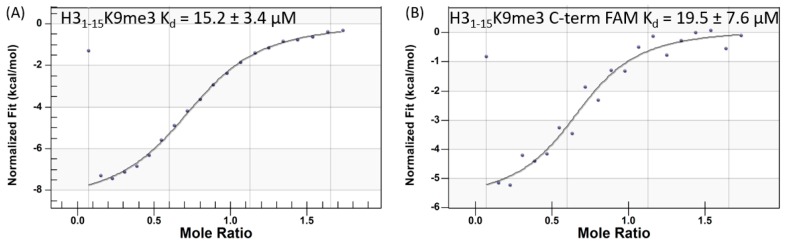
Binding affinities of H3_1-15_K9me3 (**A**) and H3_1-15_K9me3-FAM (**B**) for the ATRX_ADD_, determined via ITC.

We began our ITC studies with labelled peptides, by evaluating whether an H3_1–15_K9me3 labelled at the N-terminus with the fluorescent FAM label retained affinity for ATRX_ADD_. Interestingly, the addition of the N-terminal label completely abolished the ability of the H3 peptide to bind ATRX_ADD_ (data not shown). This initially surprising result prompted our recently reported computational studies, which clearly demonstrated that the alanine residue at the N-terminus of the H3 tail (A1) is a key anchor point for the H3:ATRX_ADD_ PPI [15]. Our computational modeling also suggested that the C-terminus of H3_1-15_ does not form any binding interactions with ATRX_ADD_; therefore, we evaluated whether FAM-labelling at this position affected affinity. As expected, the H3_1-15_K9me3 peptide incorporating a FAM-label at the C-terminus (H3_1–15_K9me3-FAM) demonstrated comparable binding affinity to ATRX_ADD_ (Figure 1), establishing this peptide as a suitable reagent for developing our fluorescence polarization (FP) assay. 

We planned to utilize an AlphaScreen assay as our orthogonal follow-up assay to our initial FP screen (vide infra). With this in mind, we also utilized ITC to verify that incorporation of a biotin label at the C-terminus of the H3 peptide did not affect the affinity for ATRX_ADD_ (Table 2). The AlphaScreen platform also requires a tag on the ATRX_ADD_ protein; therefore, we evaluated binding of our C-terminal biotin-labelled H3 peptide (H3_1–15_K9me3-biotin) to the ATRX_ADD_ domain protein, that retained the GST tag utilized for purification (ATRX_ADD_-GST). The biotinylated H3K9me3 peptide and our GST-tagged ATRX_ADD_ retained binding affinity comparable to the unlabeled components (Table 2). Following initial assay development issues using the ATRX_ADD_-GST fusion in the AlphaScreen assay (see below), we expressed and purified a His-tagged ATRX_ADD_ (ATRX_ADD_-His) protein, which also demonstrated comparable binding, verifying its suitability as a reagent for assay design.

### 2.2. Assay Development

#### 2.2.1. Fluorescence Polarization Assay

**Figure 2:**
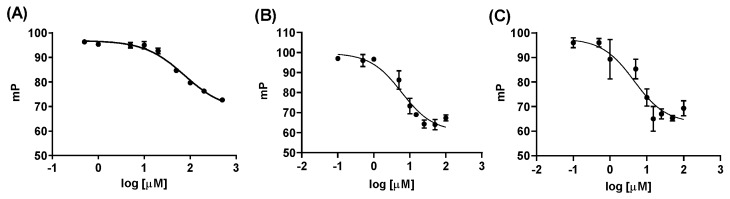
Concentration-dependent disruption of the H3:ATRX_ADD_ PPI by H3_1-15_K9me0 (**A**), H3_1-15_K9me3 (**B**), and H3_1-15_K9me3-biotin (**C**). Graphs are from a single representative experiment, performed in triplicate. All peptides were repeated at least three separate times, to provide the IC_50_ values listed in Table 1 and Table 2.

With a suitable FAM-labelled H3_1–15_ peptide in hand, we began to develop and optimize conditions for a fluorescent polarization (FP) screening assay, to more rapidly evaluate the ability of a small molecule to displace the H3:ATRX_ADD_ PPI. Initial optimization protocols focused on varying the concentrations of H3_1–15_K9me3-FAM and ATRX_ADD_ independently, to identify the concentrations of each reagent that would maximize the FP window between the bound and unbound states. Final concentrations of ATRX_ADD_ (5 µM) and H3_1–15_K9me3-FAM (0.75 μM) were then incubated in the presence of increasing concentrations of unlabeled H3_1-15_K9me3. A concentration-dependent disruption of the H3_1-15_K9me3:ATRX_ADD_ PPI by the unlabeled peptide (IC_50_ = 5.5 ± 0.2 µM, Table 1) closely mirrored the K_d_ value determined in our ITC studies. In addition, we evaluated the H3_1–15_ peptides, containing various K9 methylation patterns, as well as our biotin-labelled peptide (Table 1 and Figure 2). Overall, the IC_50_ values obtained in the FP assay were slightly lower than the corresponding K_d_ obtained through ITC; however, the established trends held, and these results, along with the calculated Z-factor (0.87), suggested that this assay was suitable for additional screening. 

#### 2.2.2. AlphaScreen Assay

Once the FP assay for screening was developed and optimized, we needed an orthogonal assay for the confirmation of any hit compounds identified from our planned screens. We decided to use Perkin Elmer’s AlphaScreen assay technology for this complementary assay, and performed an initial evaluation of its suitability, by exploring disruption of the AlphaScreen complex between H3_1-15_K9me3-biotin and ATRX_ADD_-GST. Optimization of assay parameters are well-explained in the Perkin Elmer GST Detection kit practical guide and were followed closely [16]. The first experiment involved testing an array of concentrations of each component to find the hooking point, which is the ideal concentration of each binding component (H3_1–15_K9me3-biotin and ATRX_ADD_-GST), that provides the maximum signal when bound. In other words, the hooking point provides the concentration of each reagent, at which a large signal window between bound and unbound is attained. Graphically visualizing the signal response of peptide against protein concentrations defined our hooking point at 78 nM for ATRX_ADD_-GST and 94 nM for H3_1–15_K9me3-biotin. 

Following identification of the hooking point concentrations of the two key reagents (Figure S3), we evaluated the ability of the unlabeled H3_1-15_K9me3 to disrupt the PPI in the AlphaScreen assay (Figure S4). Interestingly, when utilizing the AlphaScreen GST Detection kit, there was a significantly higher background signal than expected in the control wells, that did not include ATRX_ADD_-GST (H3_1-15_K9me3-biotin and the two beads), suggesting the histone peptide was also interacting non-specifically with the Anti-GST beads (Table 3). To investigate whether this was an interaction specific to the beads in the GST kit or a non-specific interaction across all AlphaScreen kits, we evaluated H3_1-15_K9me3-biotin in the presence of beads from the Nickel Chelate kit (Ni-NTA; Table 3). The background signal decreased significantly with the Ni-NTA beads; therefore, we continued development and optimization of the AlphaScreen assay with the Ni-NTA kit and a newly designed ATRX_ADD_-His protein. 

Hooking point concentrations for the AlphaScreen Ni-NTA assay kit were identified as 625 nM for ATRX_ADD_-His and 94 nM for H3_1–15_K9me3-biotin. Further optimization of the buffer components was also needed, to further reduce the background signal for the Ni-NTA kit. Three buffers were compared in standard control conditions, a HEPES-based buffer, phosphate buffered saline (PBS), and Perkin Elmer’s proprietary epigenetic buffer (Table S4). Across the six conditions, the Ni-NTA kit with the HEPES buffer showed the greatest signal-to-noise ratio between bound and unbound forms, and all remaining experiments were performed in this buffer. With these improved assay parameters in place, we evaluated the ability of the differentially methylated H3_1–15_ peptides for their ability to disrupt the PPI between ATRX_ADD_-His and H3_1–15_K9me3-biotin (Table 4). Overall, our results with the AlphaScreen kit closely matched those from the ITC and FP assays and provided us with a secondary orthogonal assay, that could be utilized to validate any hits identified through the primary FP screen. 

### 2.3. Screening Results

#### 2.3.1. Biochemical Screening

Our original goal, with respect to developing the assays described above, was to identify a small molecule(s) that could disrupt the H3K9me3:ATRX_ADD_ PPI for potential use as a chemical probe. To this end, we performed a medium-throughput biochemical screen of approximately 10K compounds from the ChemBridge DiverSET library in our optimized FP assay. We chose to evaluate the compounds in the FP assay, because it is more robust and reproducible than the AlphaScreen assay. Each compound was evaluated at a single concentration (10 µM) and percentage displacement was calculated based on control conditions. Small molecules that were displaced greater than 70% of the H3_1–15_K9me3-FAM from ATRX_ADD_ at this concentration were designated as initial hits (41 compounds out of the 10,240 screened, 0.4%). Each of these initial hit compounds was re-evaluated in the FP assay at several concentrations (1, 10, and 50 µM), to validate their activity in the initial screen and get a preliminary characterization of their ability to disrupt the PPI in a concentration-dependent fashion. Unfortunately, none of the initial hits demonstrated activity in the follow-up assays, suggesting they were all false positives. 

#### 2.3.2. Virtual Screening 

**Figure 3:**
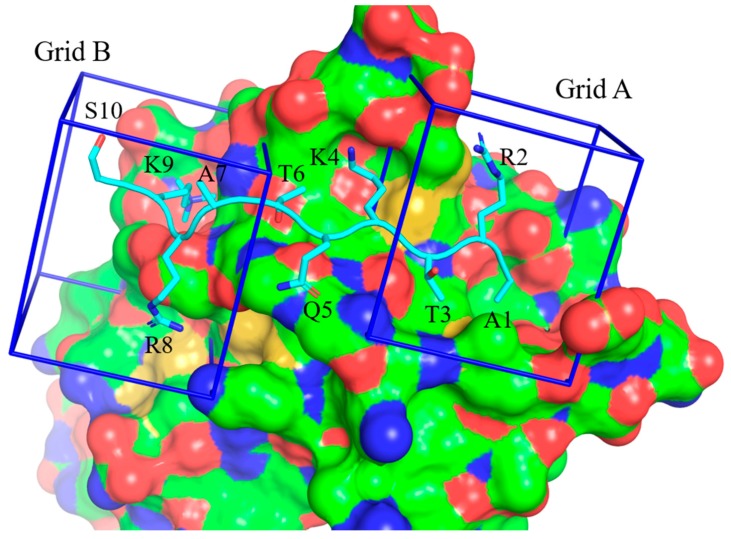
Histone H3 bound to ATRX_ADD_ with the docking sites highlighted (Modified from PDB ID 3QLA). Surface colors: red = negatively charged surface area; blue = positively charged surface area; green = hydrophobic; yellow = methionine (sulfur).

Based on our disappointing results from the biochemical screen, we decided to perform a structure-based virtual screen, with a larger database of small molecule structures. As noted above, our previous computational studies not only highlighted the importance of the H3K9 residue for high affinity H3:ATRX_ADD_ binding interactions, but they also identified A1 as an anchor residue and key contributor to the PPI. With this in mind, we utilized both binding regions on ATRX_ADD_ for our computational screen. Grid boxes were generated on the structure of ATRX_ADD_ (PDB ID 3QLA) around the binding regions of either H3A1-T3 (Grid A) or H3R8-S10 (Grid B) (Figure 3). The Schrödinger Glide software was utilized in an iterative protocol to perform separate docking studies of the same 150K small molecule database against the two distinct grid boxes on ATRX_ADD_ (Figure S5). Compounds were initially docked against the grid boxes using the high-throughput virtual screening (HTVS) protocol. Compounds with a predicted docking score ≤ −5 were re-docked into the same grid boxes, utilizing the more rigorous Glide standard precision (SP) docking procedure. Compounds that returned a Glide SP predicted binding score of ≤ −6 were subjected to a final docking procedure using the Glide extra precision (XP) mode. Compounds with a Glide XP predicted binding score of ≤ −7 were identified as potential hits. 

Based on the results of the XP docking, we chose a series of potential binders for each grid and evaluated their ability to disrupt the H3:ATRX_ADD_ PPI in our FP assay (Tables S5 and S6). Similar to our results from the biochemical screening, none of these predicted hits from the virtual screen were active at disrupting the PPI in the FP assay, even up to concentrations of 100 µM (data not shown). We also evaluated various combinations of our top hits from both grids, to determine whether combined disruption at both sites could disrupt the PPI. Unfortunately, none of these combinations were active, suggesting that either the virtual screen was ineffective at identifying compounds that could bind to these regions on ATRX_ADD_, or that unlinked small molecules are not capable of disrupting the multiple anchor points associated with the H3:ATRX_ADD_ PPI, to the extent required for the complete displacement of the H3 peptide.

### 2.4. Truncated H3 Peptide Studies

In parallel to our attempts to identify small molecules capable of disrupting the H3:ATRX_ADD_ PPI, we utilized the higher-throughput FP assay to further explore the structural requirements for the binding of H3 to the ATRX_ADD_ domain. We evaluated multiple truncated and modified H3 peptides to more fully understand which residues are essential for binding to ATRX_ADD_ (Table 5 and Table S1). Overall, the results from the truncated peptides evaluated in the FP assay further validated the results of our computational studies that the A1, K4, and K9 residues are key anchor points for H3 binding to ATRX_ADD_. The only peptides that were able to completely displace the FAM H3_1-15_K9me3 peptide from ATRX_ADD_ had at least 10 amino acid residues, all three anchor residues, and the ADD domain specific methylation patterns at K4 and K9 (me0 and me3, respectively). Truncated peptides that contained only one anchor residue (H3_1-5_, H3_3-5_, H3_2-6_, H3_3-8_, H3_7-11_K9me3) were inactive. 

The evaluation of truncated peptides that contained two of the anchor residues also provided important insight into H3:ATRX_ADD_ binding. Removing the A1 anchor completely abolished activity (H3_3-11_K9me3), highlighting the importance of contacts in this region for high affinity binding. Interestingly, replacing the A1 residue with slightly bulkier, hydrophobic residues (H3_1-11_ A1V and H3_1-11_ A1L) was well-tolerated, suggesting that the A1 binding pocket is amenable to other side chains. Overlays of each of these peptides docked into ATRX_ADD_ clearly demonstrates that the A1 side chain does not occupy the entire pocket upon binding (Figure S6). ‘Removing’ the unmodified K4 anchor by masking it as K4me3 (H3_1-11_K4me3K9me3) significantly reduced the activity. The truncated removal of the K9 anchor resulted in a peptide (H3_1-8_) that was unable to disrupt the H3:ATRX_ADD_ PPI. Finally, the inability of a combination of two peptides (H3_1-5_ + H3_7-11_K9me3) containing all three anchor residues to displace H3_1-15_K9me3, clearly demonstrates that the hot spot residues must be intact in a single structure to disrupt the PPI. 

## 3. Discussion

The ATRX_ADD_ demonstrates a unique affinity for the H3 tail, but only when K4 is non-modified and K9 is trimethylated [11,12]. Our previous computational studies also suggested an important binding role for the N-terminal alanine residue of H3. Our goals for the studies reported herein were two-fold. First, we sought to develop biochemical assays with a higher throughput compared to ITC that could be utilized to experimentally validate our computational results. Second, we wanted to utilize these assays in small molecule screening efforts to identify compounds that disrupt the H3:ATRX_ADD_. These compounds would be useful probes to more fully explore how loss-of-function of the ATRX_ADD_ contributes to oncogenic transformation. 

Initial optimization of the FP assay was straightforward and provided a robust assay that can be rapidly utilized to explore this PPI. By contrast, development of the AlphaScreen assay was met with only modest success. This assay required significant optimization to get an acceptable change in signal between bound and unbound conditions for both the GST and Nickel chelate kits. Interestingly, the loss of signal when the proprietary epigenetics buffer was used suggests that binding in this assay might be reliant on the charge difference between the protein and the peptide. This could be further investigated by using either a longer sequence of the H3 tail or purified nucleosomes; however, these modifications would complicate the assay design and reduce its potential as a suitable orthogonal assay for studying the PPI.

While there are discrepancies between the absolute K_d_ and IC_50_ values determined across the various experimental protocols, these values do not represent a significant pattern change. Across all our assays, H3K9me0 is the weakest binder. In general, increasing the methylation state at K9 enhances the binding affinity and activity of the peptide. H3K9me2 and H3K9me3 are significantly more active in the ITC, FP, and AlphaScreen assays and we see the absolute IC_50_ values of these peptides as comparable across the assays (low micromolar inhibition). Our results also track very well with those previously reported, i.e., that the di- and trimethylated H3 peptides are comparable in activity and significantly more active than the unmodified and mono-methylated peptides.

Our studies in the FP assay with the truncated H3 peptides provide essential details about the binding mechanism(s) that govern the high affinity, specific binding between ATRX_ADD_ and the H3 tail. Incorporation of the FAM label on the N-terminus of the H3 peptide completely abrogated its ability to bind to ATRX_ADD_, highlighting the importance of a free N-terminus on H3. Removal of the A1 anchor also resulted in an inactive peptide. Taken together, these data support our previous molecular dynamics simulations, which suggested that the A1 anchor contributes a majority of the H3 binding energy for ATRX_ADD_ [15]. Interestingly, small modifications to the side chain of the A1 anchor were well-tolerated, suggesting the binding pocket for the alanine side chain is amenable to minor changes, as long as the hydrophobic nature of the residue is maintained. Our results also fully support previous studies demonstrating that intermolecular interactions between the K4/K9me3 residues and ATRX_ADD_ are essential for high affinity binding. 

In parallel with our binding studies, we undertook biochemical and computational screening approaches to identify small molecules capable of disrupting the H3:ATRX_ADD_. Both of these strategies were unsuccessful at identifying a positive hit; this result is not surprising when our previous computational studies and our current truncated peptide displacement studies are considered. The presence of all three anchor residues (A1, K4, and K9) in a single peptide was required for disrupting the PPI in our FP assay. These results strongly suggest that a single small molecule targeting one of these binding regions will not be capable of occupying all three binding pockets in a manner that will completely displace the H3 tail.

## 4. Materials and Methods 

### 4.1. General Information

All peptides were purchased from GenScript. Plasmids for the expression of ATRX_ADD_-GST were obtained from Addgene. All reagents and chemicals were from Fisher Scientific or Sigma Aldrich (USA), unless otherwise noted. Fluorescence polarization assays were performed on a BioTek Synergy H1 Hybrid Reader. The AlphaScreen assays were performed on an Envision Multimode Plate Reader (New Haven, Connecticut, USA).

### 4.2. Plasmids and Cloning

The pGEX-GP-2-ATRX ADD plasmid (Addgene plasmid #59698) [17] was transformed into BL21 cells for expression and purification, using the GST tag in the pGEX-4T1 vector. The His-tagged ADD plasmid was cloned using the following primers: forward (GCTATCCATATGAGCTGCACTGCTTGTGGA) and reverse (GCTATCGGATCCTCACTGCAACAACTGTTCTAAATTCTC). The PCR protocol for amplification of the ADD insert had an annealing temperature of 56.4 °C for 30 seconds, with an extension time of 30 seconds. The PCR product was gel purified and double digested with NdeI and BamHI for 4 hours at 37 °C. The double digest product was gel purified and ligated for 18 hours at 16 °C, with the double digested pET-15b vector. The ligation product was transformed into competent DH5α cells and a single isolated colony was sequenced. After successful sequencing, the plasmid DNA was re-transformed into BL21 cells.

### 4.3. Protein Expression and Purification

The transformed BL21 cells were grown at 37 °C to OD_600_ ≈ 0.6, then induced overnight at room temperature with 1 mM IPTG. Cells were pelleted and frozen at −20 overnight to help with the lysis. While thawing, cells were resuspended in a Lysis buffer containing 20 mM Tris (pH 7.5), 400 mM KCl, a complete EDTA-free Protease Inhibitor cocktail tablet, and a dash of lysozyme. The His-tagged ATRX_ADD_ required the addition of BugBuster Protein Extraction Reagent (~25%) to the lysis buffer to solubilize the protein. After resuspension, sonication was used to lyse the cells. The supernatant was concentrated and loaded onto Pierce Spin Columns (Glutathione or Ni-NTA) and nutated for 1 hour at 4 °C. After washing, ATRX_ADD_-GST or ATRX_ADD_-His could be eluted with the elution buffer from the kits protocol or on column cleavage could be performed using Thrombin (bovine, MP Biomedicals), 60 U/1 L induced cell growth, nutating overnight at 4 °C, and then eluted with wash buffer as described in the spin column protocol. Washes were monitored by Bradford reagent and quantified by measuring absorbance. The collected protein was concentrated and buffer was exchanged into the storage buffer [20 mM Tris (pH 7.5), 400 mM KCl, and 5 mM DTT]. Following affinity chromatography, ATRX_ADD_-His was purified via FPLC, with a Superdex 75 Gel filtration column (Storrs, Connecticut, USA). 

### 4.4. Isothermal Titration Calorimetry

The ITC experiments were performed on a Nano-ITC Calorimeter (TA instruments, Storrs, Connecticut, USA). The ATRX_ADD_ was buffer exchanged from the storage buffer (20 mM Tris (pH 7.5), 400 mM KCl, and 5 mM DTT) to the test buffer (20 mM Tris (pH 7.5), 400 mM NaCl, 5 mM β-mercaptoethanol (BME)), using Amicon Ultra-2 Centrifugal Filter Devices. All experiments were performed at 25 °C, with a titration of 2.5 μL of H3 peptide every 300 seconds. Calorimetric data was analyzed using NanoAnalyze Software modeling with an independent fit and a blank (constant) fit to provide the noted K_d_ values.

### 4.5. Microscale Thermophoresis

The MST experiments were performed on a NanoTemper Monolith NT.115 using the RED-NHS protein labelling kit from NanoTemper (Cambridge, Massachusetts, USA), which labels the lysines in the protein (ATRX_ADD_). ATRX_ADD_ was buffer exchanged from the storage buffer (20 mM Tris (pH 7.5), 400 mM KCl, and 5 mM DTT) to the test buffer (20 mM HEPES, (pH 7.5), 100 mM NaCl, and 2 mM TCEP) using Amicon Ultra-2 Centrifugal Filter Devices. The protein was labelled and purified following the kit protocol. The protein concentration and degree of labelling was calculated based off A_205_ and A_650_, following the equations in the manual for the labelling kit. Expert mode in the MO Control software was used for all runs due to solubility of peptides. The peptides were all dissolved in 100% DMSO and a dilution set of 16 concentrations from 10 mM to 200 nM was used to make the final reaction mixtures with 19 μL of labelled ATRX_ADD_ (200 nM, diluted in the test buffer with 0.005% Tween 20) and 1 μL of the peptides in DMSO; 10 μL of this reaction was loaded into standard capillaries. MO Affinity Analysis software was used to analyze the MST trace for binding affinity.

### 4.6. Fluorescence Polarization Assay

ATRX_ADD_ (20 μM, 5 μL) and H3_1-15_K9me3-FAM (3 μM, 5 μL) were diluted in phosphate buffered saline. These two components (total 10 μL) were incubated for 15 minutes at room temperature in a black 384-well plate (ProxiPlate-384 F Plus, PerkinElmer, Waltham, Massachusetts, USA), prior to adding the H3 peptide or small molecule. Unlabeled peptides or small molecules (10 μL, varying concentrations) were added, to give the ATRX_ADD_ and FAM labelled-peptide final concentrations of 5 μM and 0.75 μM in 20 μL total volume per well. All components were diluted in sterile PBS. 

### 4.7. Alphascreen Assay

#### 4.7.1. Initial Assay Development

Initial assay development followed Perkin Elmer’s (Waltham, Massachusetts, USA)practical guide for AlphaScreen assays [16]. The total reaction volume is 10 μL with 20 μg/mL of each bead, and the concentrations of labelled ATRX_ADD_ and H3_1-15_K9me3-biotin were optimized using an array, to identify the hooking point as discussed in the practical guide. Once the optimal concentrations of each binding component were determined, the controls were run to observe the degree of background signal. It was at this point that the high background with H3_1-15_K9me3-biotin and both beads (Streptavidin and Anti-GST) was higher than expected. This prompted our optimization with the Ni-NTA kit instead of the Anti-GST kit. After proper binding confirmation with purified ATRX_ADD_-His, optimization was repeated with the Ni-NTA AlphaScreen assay kit and the following three buffers: (1) HEPES buffer (50 mM HEPES, 100 mM NaCl, 0.1% Triton X-100, 0.1% BSA), (2) PBS, and (3) Perkin Elmer’s proprietary epigenetics buffer. 

#### 4.7.2. Optimized Assay Protocol

ATRX_ADD_-His (625 nM) and H3_1-15_K9me3-biotin (94 nM), combined with 20 μg/mL of each bead (donor and receptor), were diluted in the HEPES buffer (total volume of 4 components = 6 μL). The peptide or compound being used to interrupt the PPI was diluted in the HEPES buffer manner (4 μL), to bring the final volume in the well to 10 μL. The reaction was incubated in the dark at room temperature for 1 hour and then read on an Envision Multimode Plate Reader. 

### 4.8. Biochemical Screening

A subset of compounds (10,240) from the ChemBridge DiverSET library were evaluated for their ability to disrupt the H3:ATRX_ADD_ PPI, using the optimized FP assay protocol described above. All compounds were evaluated at 10 µM and the DMSO concentration in each well was 0.5%. The screening data was normalized based on the controls to calculate a percent displacement value. Promising hits (≥ 70% displacement) from the initial screen were re-evaluated at three concentrations (1 μM, 10 μM, and 50 μM).

### 4.9. Virtual Screening

Schrodinger Maestro 10.5 was used for grid generation and docking. Two grids were generated using a centroid of mass around residues A1, R2, T3 (Grid A) or R8, K9, S10 (Grid B) of the H3 peptide, because there is no ligand bound to the ATRX_ADD_ in the crystal structure 3QLA. Grid A was a 25 Å cube and Grid B was a 30 Å cube. Following grid generation, the imported ligands from the small molecule database were prepared using the OPLS3 force field. The prepared ligands were docked in each of the grids, using high throughput virtual screening, standard precision docking, and extra precision docking (allowing for 10 conformations per ligand in XP mode). Hits from the virtual screen were tested in the fluorescence polarization assay, first individually, and then in combination studies with a compound hit from each grid at 10 μM and 20 μM.

## Figures and Tables

**Table 1:** In vitro binding affinity and IC_50_ displacement for ATRX_ADD_ and various H3 peptides.

H3 Peptide	K_d_ (µM) ^1^, ITC	K_d_ (µM) ^2^, ITC	K_d_ (µM) ^3^, MST	IC_50_ (µM) ^4^, FP
H3_1–15_K9me0	7.9	369 ± 47	23.4 ± 11	76.8 ± 1.3
H3_1–15_K9me1	2.5–2.9	289 ± 2.4	3.4 ± 2.2	63.3 ± 9.5
H3_1–15_K9me2	0.38–1.3	49.5 ± 8.6	4.9 ± 2.6	10.2 ± 1.0
H3_1–15_K9me3	0.27–0.5	15.2 ± 3.4	1.8 ± 0.2	5.5 ± 0.2
H3_1–15_K9me3-FAM	ND ^5^	19.5 ± 7.6	ND	ND

^1^ K_d_ values ranges determined by ITC reported in references 11 and 12. ^2^ K_d_ values determined by ITC for this manuscript are the average of at least two separate experiments. ^3^ K_d_ values determined by MST for this manuscript are the average of at least two separate experiments. ^4^ IC_50_ values determined via the FP assay are the average of at least three separate experiments performed in triplicate. ^5^ ND = Not Determined.

**Table 2:** In vitro binding affinity and IC_50_ displacement for tagged ATRX_ADD_ and H3_1-15_K9me3 biotin.

Tagged ATRX_ADD_	H3 Peptide	K_d_ (μM) ^1^	IC_50_ (μM) ^2^
ATRX_ADD_-GST	H3_1–15_K9me3-biotin	37.6 ± 0.1	5.3 ± 0.1
ATRX_ADD_-His	H3_1–15_K9me3-biotin	14.3 ± 2.7	2.8 ± 0.8

^1^ K_d_ values determined by ITC are the average of at least two separate experiments. ^2^ IC_50_ values determined via the FP assay are the average of at least three separate experiments performed in triplicate.

**Table 3:** H3_1-15_K9me3-biotin binds non-specifically to the AlphaScreen GST beads.

Peptide	Protein	Beads	Signal ^1^
H3_1–15_K9me3-biotin	ATRX_ADD_-GST	Anti-GST	722400 ± 33000
H3_1–15_K9me3-biotin	---	Anti-GST	224400 ± 14000
H3_1–15_K9me3-biotin	ATRX_ADD_-GST	Ni-NTA	5218 ± 80
H3_1–15_K9me3-biotin	---	Ni-NTA	5778 ± 220
H3_1–15_K9me3-biotin	ATRX_ADD_-His	Ni-NTA	1246000 ± 58000
H3_1–15_K9me3-biotin	---	Ni-NTA	1260 ± 430

^1^ Signal values are the average of at least two separate experiments.

**Table 4:** Disruption of the H3_1-15_K9me3-biotin:ATRX_ADD_-His PPI by H3 peptides.

Peptide	Beads	IC_50_ (μM)
H3_1–15_K9me0	Ni-NTA	> 10.000 ^1^
H3_1–15_K9me1	Ni-NTA	> 10.000
H3_1–15_K9me2	Ni-NTA	3.9 ± 2.5
H3_1–15_K9me3	Ni-NTA	11.6 ± 0.3

^1^ IC_50_ values determined via the AlphaScreen assay are the average of at least two separate experiments performed in triplicate.

**Table 5:** Disruption of the H3_1-15_K9me3:ATRX_ADD_ PPI by modified H3 peptides.

Peptide	Truncated Peptide Sequence	FP Assay IC_50_
H3_1-16_	ARTKQTARKSTGGKAY	85.8 ± 19.3 μM
H3_1-10_	ARTKQTARKS	94.8 ± 4.6 μM
H3_1-5_	ARTKQ	ND ^1^
H3_3-5_	TKQ	ND
H3_2-6_	RTKQT	ND
H3_3-8_	TKQTAR	ND
H3_1-11_K9me3	ARTKQTARK(me3)ST	7.6 ± 1.4 μM
H3_7-11_K9me3	ARK(me3)ST	ND
H3_1-5_ + H3_7-11_K9me3	ARTKQ + ARK(me3)ST	ND
H3_1-7_K4me3	ARTK(me3)QTA	ND
H3_3-10_K4me3K9me3	TK(me3)QTARK(me3)S	ND
H3_1-11_ A1V	VRTKQTARK(me3)ST	4.3 ± 0.8 μM
H3_1-11_ A1L	LRTKQTARK(me3)ST	5.05 ± 0.7 μM
H3_1-8_	ARTKQTAR	≥ 100 μM ^2^
H3_3-11_K9me3	TKQTARK(me3)ST	ND
H3_1-11_K4me3K9me3	ARTK(me3)QTARK(me3)ST	≥ 100 μM

^1^ ND = no displacement. ^2^ Modest displacement (~30%) was observed at the highest concentration tested (100 µM), but an IC_50_ value could not be calculated.

## Supplementary Materials

The following are available online at https://www.mdpi.com/1420-3049/25/7/1500/s1, Figure S1: Binding affinities for H3_1-15_ peptides bound to ATRX_ADD_ as determined by microscale thermophoresis (MST), Table S1: Parallel Fluorescence Intensity Signals from FP Optimization, Figure S2: Assay window from FP optimization studies, Table S2: Relative Luminescence Values from AlphaScreen Optimization, Figure S3: Assay window from AlphaScreen optimization studies, Figure S4: Disruption of the ATRX_ADD_:H3 PPI under various conditions through the AlphaScreen assay, Figure S5: Schematic of workflow for virtual screening using Schrodinger Glide, Figure S6: Modeling of H3 peptides into the ATRX_ADD,_ Table S3: Amino acid sequences for peptides utilized in the FP and AlphaScreen optimization, Table S4: Results for Ni-His AlphaScreen Buffer Optimization, Table S5: Virtual Screening Predicted Hits for Grid A (ART), Table S6: Virtual Screening Predicted Hits for Grid B (RKS).

## References

[1] Gibbons R.J., Picketts D.J., Villard L., Higgs D.R. (1995). Mutations in a putative global transcriptional regulator cause X-linked mental retardation with α-thalassemia (ATR-X syndrome). Cell.

[2] Li Z., Zhao D., Xiang B., Li H. (2017). Structural and biochemical characterization of DAXX-ATRX interaction. Protein Cell.

[3] Hoelper D., Huang H., Jain A.Y., Patel D.J., Lewis P.W. (2017). Structural and mechanistic insights into ATRX-dependent and -independent functions of the histone chaperone DAXX. Nat. Commun.

[4] Szenker E., Ray-Gallet D., Almouzni G. (2011). The double face of the histone variant H3.3. Cell Res..

[5] Ratnakumar K., Bernstein E. (2013). Atrx. Epigenetics.

[6] Koschmann C., Calinescu A.-A., Nunez F.J., Mackay A., Fazal-Salom J., Thomas D., Mendez F., Kamran N., Dzaman M., Mulpuri L. (2016). ATRX loss promotes tumor growth and impairs nonhomologous end joining DNA repair in glioma. Sci. Transl. Med..

[7] Gibbons R.J., Wada T., Fisher C.A., Malik N., Mitson M.J., Steensma D.P., Fryer A., Goudie D.R., Krantz I.D., Traeger-Synodinos J. (2008). Mutations in the chromatin-associated protein ATRX. Hum. Mutat..

[8] Clynes D., Higgs D.R., Gibbons R.J. (2013). The chromatin remodeller ATRX: a repeat offender in human disease. Trends Biochem. Sci..

[9] Jiao Y., Killela P.J., Reitman Z.J., Rasheed A.B., Heaphy C.M., de Wilde R.F., Rodriguez F.J., Rosemberg S., Oba-Shinjo S.M., Nagahashi Marie S.K. (2012). Frequent ATRX, CIC, FUBP1 and IDH1 mutations refine the classification of malignant gliomas. Oncotarget.

[10] Watson L.A., Goldberg H., Bérubé N.G. (2015). Emerging roles of ATRX in cancer. Epigenomics.

[11] Iwase S., Xiang B., Ghosh S., Ren T., Lewis P.W., Cochrane J.C., Allis C.D., Picketts D.J., Patel D.J., Li H. (2011). ATRX ADD domain links an atypical histone methylation recognition mechanism to human mental-retardation syndrome. Nat. Struct. Mol. Biol..

[12] Eustermann S., Yang J.-C., Law M.J., Amos R., Chapman L.M., Jelinska C., Garrick D., Clynes D., Gibbons R.J., Rhodes D. (2011). Combinatorial readout of histone H3 modifications specifies localization of ATRX to heterochromatin. Nat. Struct. Mol. Biol..

[13] Teske K.A., Hadden M.K. (2017). Methyllysine binding domains: Structural insight and small molecule probe development. Eur. J. Med. Chem..

[14] Dhayalan A., Tamas R., Bock I., Tattermusch A., Dimitrova E., Kudithipudi S., Ragozin S., Jeltsch A. (2011). The ATRX-ADD domain binds to H3 tail peptides and reads the combined methylation state of K4 and K9. Hum. Mol. Genet..

[15] Dash R.C., Zaino A.M., Hadden M.K. (2018). A metadynamic approach to understand the recognition mechanism of the histone H3 tail with the ATRXADD domain. Biochimica et Biophysica Acta (BBA) - Gene Regulatory Mechanisms.

[16] (2004). AlphaScreen^TM^: Sensitive Homogeneous Assay Technology.

[17] Jeltsch A. Addgene: pGEX-GP-2-ATRX ADD. https://www.addgene.org/59698/#how-to-cite.

