# Supplementary material for: Probing the Protein-Protein Interaction between the ATRXADD Domain and the Histone H3 Tail
Source: Molecules. 2020 Mar 25;25(7):1500. doi: 10.3390/molecules25071500 (PMC7181051; doi:10.3390/molecules25071500)
Supplement: Supplementary file 1 [file molecules-25-01500-s001.pdf]

Probing the Protein-Protein Interaction Between the $ATRX_{ADD}$ Domain and the Histone H3 Tail

Angela M. Zaino[1], Radha, C. Dash[1], and M. Kyle Hadden[1,*]

[1]Department of Pharmaceutical Sciences, University of Connecticut, 69 North Eagleville Rd, Storrs, CT, USA, 06029-3092

*Correspondence: kyle.hadden@uconn.edu; Tel.: 1-860-486-8446

Supplementary Information: Additional Figures and Tables

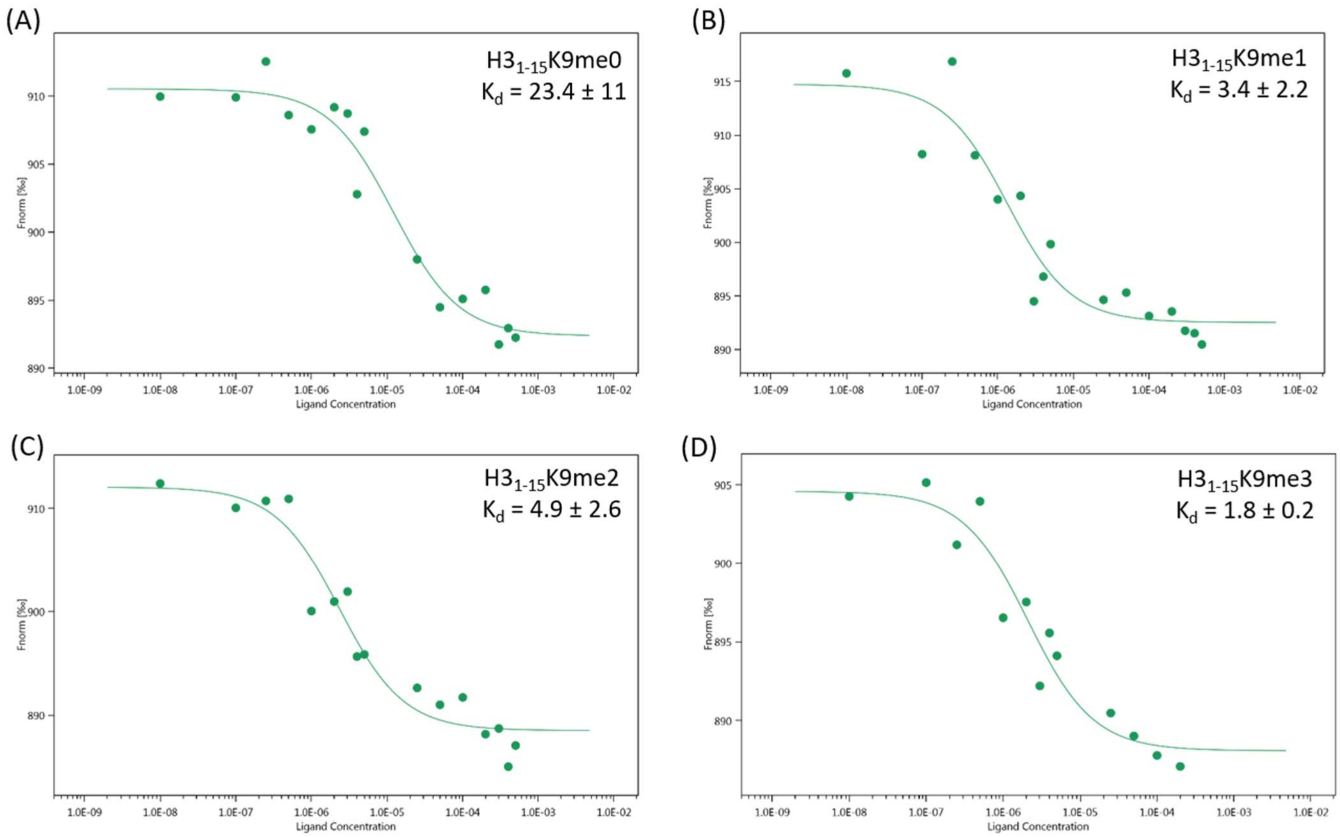

Supplementary Figure 1. Binding affinities for $H3_{1\text{-}15}$ peptides bound to $ATRX_{ADD}$ as determined by microscale thermophoresis (MST).

Supplementary Table 1. Parallel Fluorescence Intensity Signals from FP Optimization.[a]

$ATRX_{ADD}$	K9me3-FAM [μM]							
[μM]	1.5	0.75	0.375	0.188	0.094	0.047	0.023	0.012
10	23692[b]	11480	6132	3446	1720	741	387	204
5	23461	12312	6273	3474	1731	868	481	238
2.5	25637	14302	7752	4174	1762	1055	486	272
1.25	33923	18238	9213	4799	2295	1178	562	304
0.625	42374	20899	10786	5013	2342	1230	557	311
0.313	41074	20530	7903	4186	2258	1047	523	283
0.156	37005	19837	8980	4189	2298	1135	572	291
0.078	26783	20726	8824	4339	2383	1152	553	301
0	27960	8746	2011	1979	680	314	187	119

[a]All concentration combinations evaluated provided a signal >10X the buffer background (Ave Value = 19) per Reference 1.

[b]Values are the average of three separate parallel fluorescence signals obtained at each combination of $ATRX_{ADD}$ and K9me3-FAM.

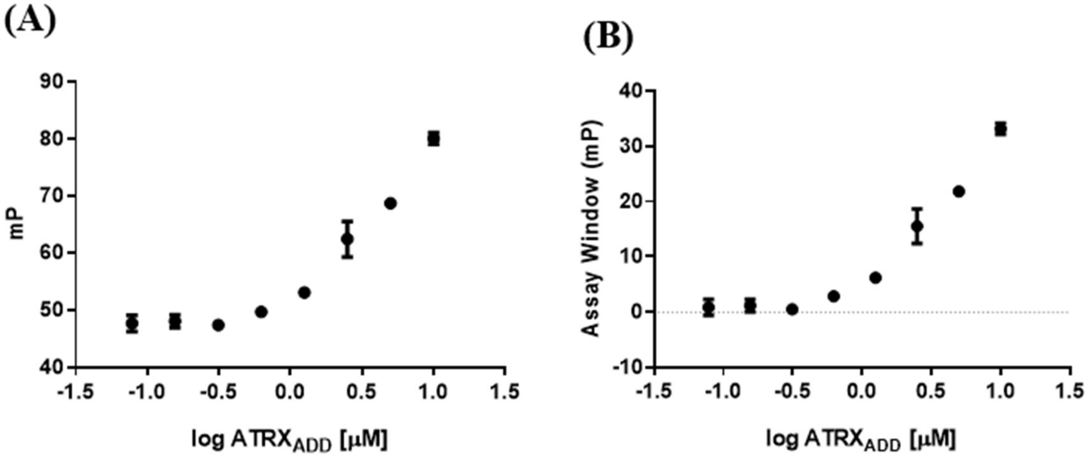

Supplementary Figure 2. Assay window from FP optimization studies. Assay windows in absolute mP (A) and adjusted mP (B) for 0.75 μM K9me3-FAM when titrated against increasing concentrations of $ATRX_{ADD}$.

Supplementary Table 2. Relative Luminescence Values from AlphaScreen Optimization.[a]

$ATRX_{ADD}$-His [μM]	K9me3-Biotin [μM]							
	1.5	0.75	0.375	0.188	0.094	0.047	0.023	0.012
10	1432611[b]	1287002	1390993	1435515	1462069	1957687	2109875	2433757
5	1716235	1649016	1789896	1809908	2093087	2475457	2518030	2456588
2.5	1958028	1961109	2085796	2108049	2287965	2713212	2609933	2609838
1.25	2369047	2229987	2350416	2248196	2408041	2706550	2707118	2499822
0.625	2651483	2541458	2630063	2559682	2649426	2503875	2203301	1189371
0.313	1402899	1893262	2057373	2129765	1879438	917692	310018	57825
0.156	330648	531409	816336	1060918	663631	181141	80803	20148
0.078	89079	150817	251388	411531	345134	112539	40979	11965
0	36587	31729	33432	31962	20887	12444	14333	13078

[a]All concentration combinations evaluated provided a signal >10X the buffer background (Ave Value = 20,542) per Reference 1.

[b]Values are the average of three separate parallel fluorescence signals obtained at each combination of $ATRX_{ADD}$-His and K9me3-Biotin.

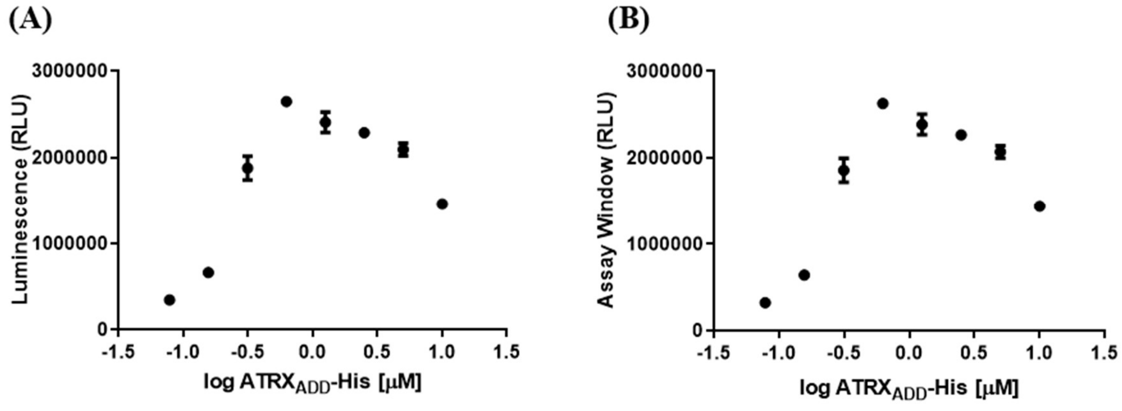

Supplementary Figure 3. Assay window from AlphaScreen optimization studies. Assay windows in absolute RLU (A) and adjusted RLU (B) for 0.094 μM $H3_{1\text{-}15}$K9me3-biotin when titrated against increasing concentrations of $ATRX_{ADD}$ provides the hooking point for further studies.

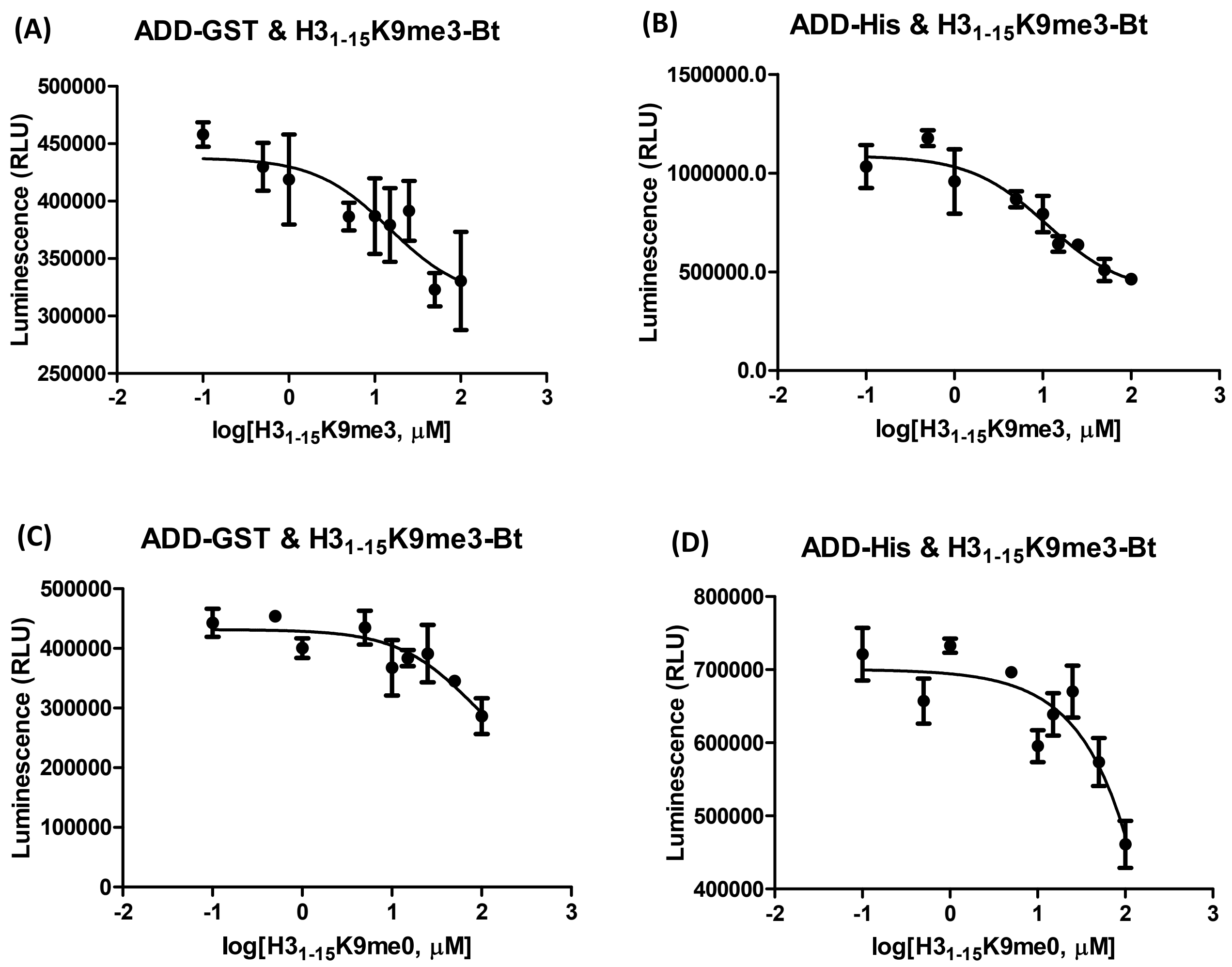

Supplementary Figure 4. Disruption of the $ATRX_{ADD}$:H3 PPI under various conditions through the AlphaScreen assay. Concentration-dependent displacement of $H3_{1\text{-}15}$K9me3-biotin from ADD-GST (A) and ADD-His (B) by $H3_{1\text{-}15}$K9me3. Concentration-dependent displacement of $H3_{1\text{-}15}$K9me3-biotin from ADD-GST (C) and ADD-His (D) by $H3_{1\text{-}15}$K9me0.

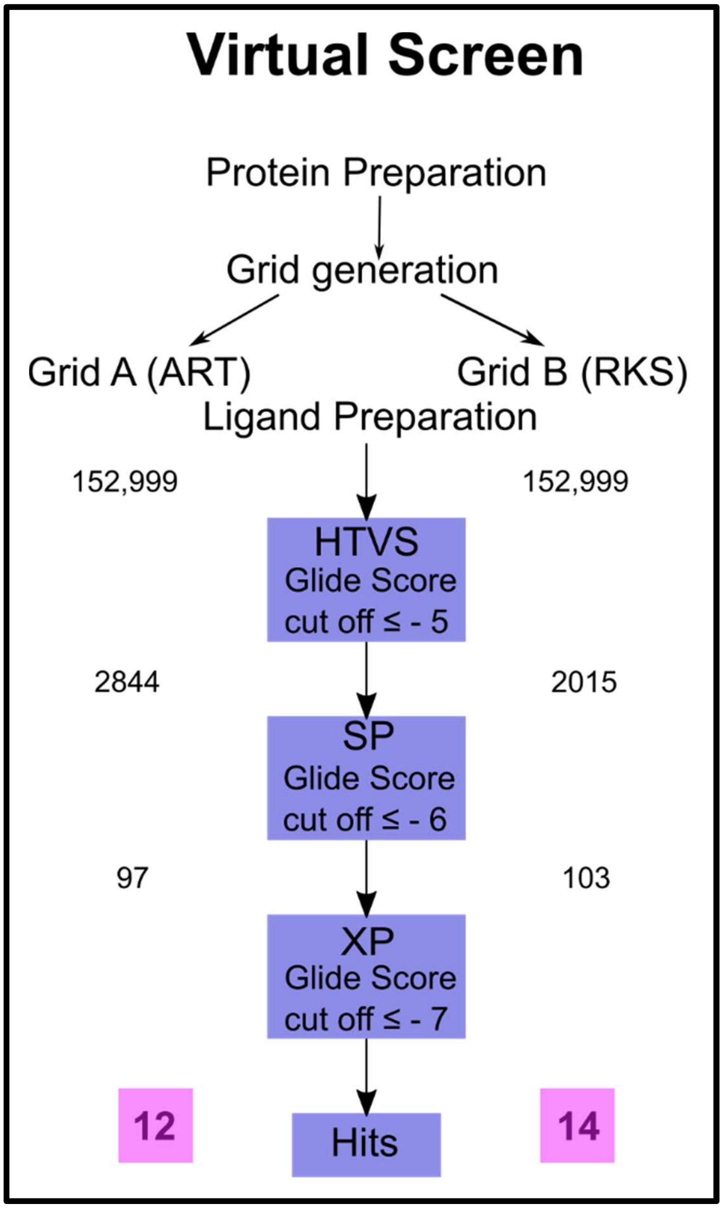

Supplementary Figure 5. Schematic of workflow for virtual screening using Schrodinger Glide

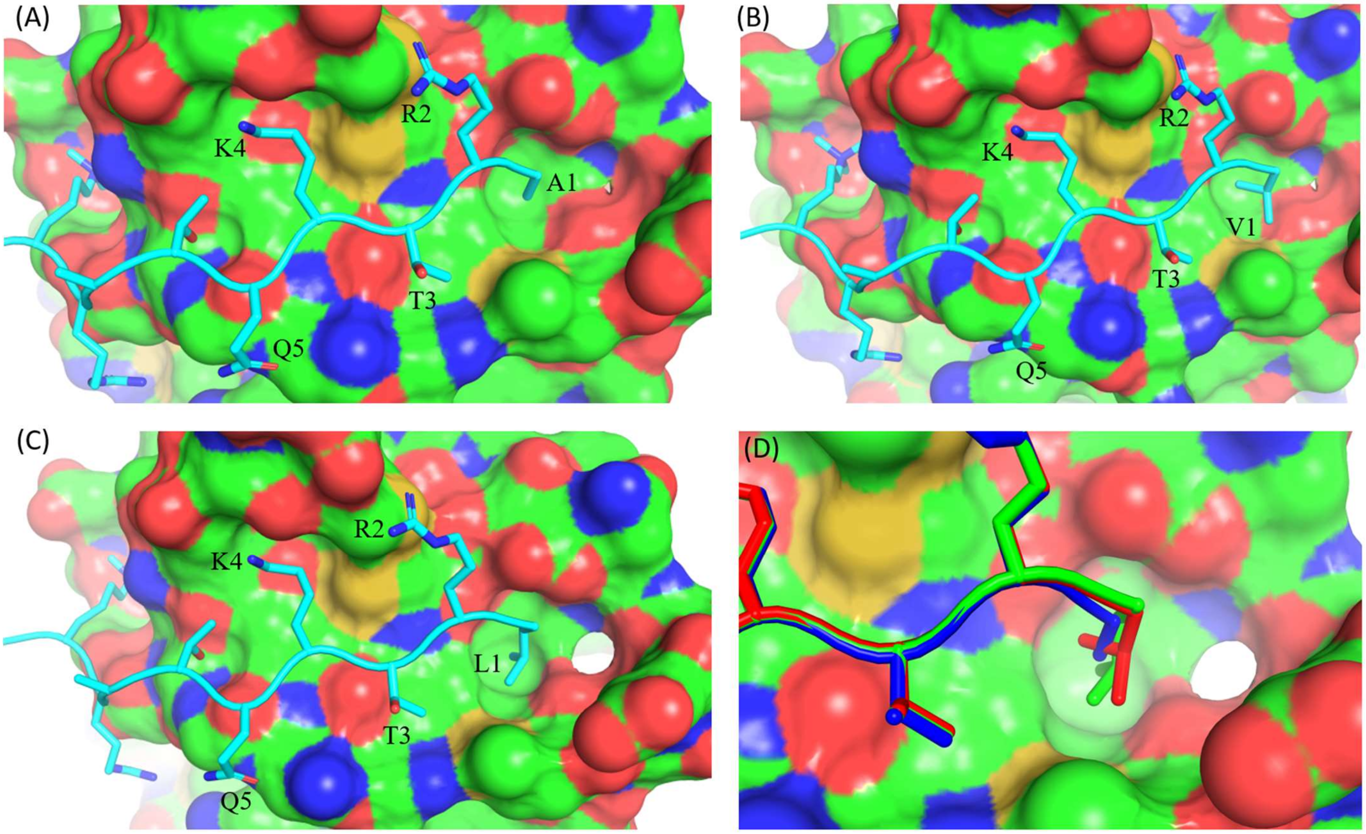

Supplementary Figure 6. Modeling of H3 peptides into the $ATRX_{ADD}$. Wild-type H3 (A), A1V (B), and A1L (C) docked into the A1 pocket (structures minimized in Maestro). The three peptides were overlaid and focused (D) on the alanine (blue), valine (red), and leucine (green) residues. Surface colors: red = negatively charged surface area; blue = positively charged surface area; green = hydrophobic; yellow = methionine (sulfur).

Supplementary Table 3. Amino acid sequences for peptides utilized in the FP and AlphaScreen optimization.

Peptide ID	Complete Sequence
$H3_{1-15}$K9me0	ARTKQTARKSTGGKA
$H3_{1-15}$K9me1	ARTKQTARKme1STGGKA
$H3_{1-15}$K9me2	ARTKQTARKme2STGGKA
$H3_{1-15}$K9me3	ARTKQTARKme3STGGKA
FAM-$H3_{1-15}$K9me3	5-FAM-ARTKQTARKme3STGGKA
$H3_{1-15}$K9me3-FAM	ARTKQTARKme3STGGKAK(ε-NH-5-FAM)}
$H3_{1-15}$K9me3-biotin	ARTKQTARKme3STGGKAK(ε-NH-biotin)

Supplementary Table 4. Results for Ni-His AlphaScreen Buffer Optimization.

Peptide	Protein	Beads	Buffer[1]	Signal
$H3_{1-15}$K9me3-biotin	$ATRX_{ADD}$-His	Ni-NTA	HEPES	582900 ± 19000
$H3_{1-15}$K9me3-biotin	---	Ni-NTA	HEPES	3403 ± 820
$H3_{1-15}$K9me3-biotin	$ATRX_{ADD}$-His	Ni-NTA	PBS	802300 ± 6400
$H3_{1-15}$K9me3-biotin	---	Ni-NTA	PBS	13860 ± 1200
$H3_{1-15}$K9me3-biotin	$ATRX_{ADD}$-His	Ni-NTA	PE epi	81640 ± 7700
$H3_{1-15}$K9me3-biotin	---	Ni-NTA	PE epi	3948 ± 110

[1]HEPES buffer: HEPES, NaCl, Triton, BSA. PBS: phosphate buffered saline. PE epi: Perkin Elmer's proprietary epigenetics buffer

Supplementary Table 5. Virtual Screening Predicted Hits for Grid A (ART).

Grid A		
Compound	Structure	XP Glide Score(s)
**1**	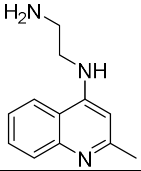	-7.718<br>-7.714
**2**	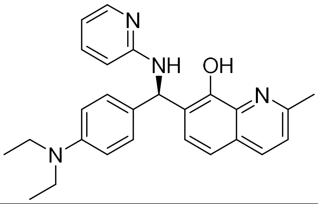	-7.749<br>-7.679<br>-7.543<br>-7.305
**3**	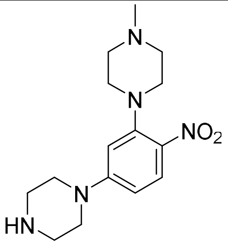	-7.633<br>-7.185
**4**	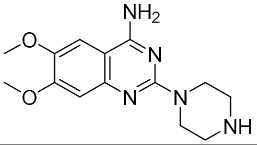	-7.53
**5**	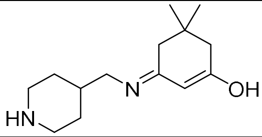	-7.397
**6**	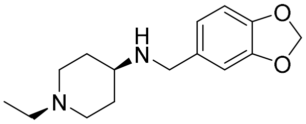	-7.385
**7**	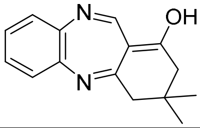	-7.38
**8**	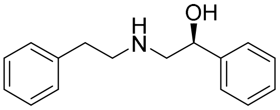	-7.334
**9**	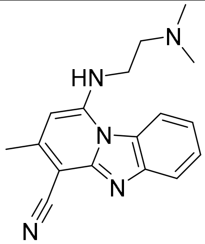	-7.291
**10**	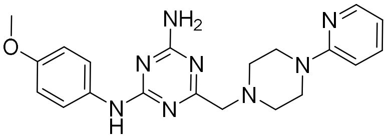	-7.175

**11**	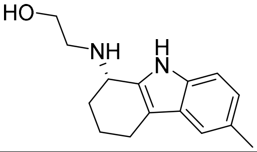	-7.103
**12**	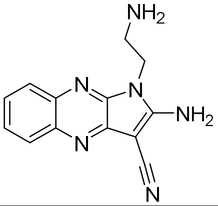	-7.027

Supplementary Table 6: Virtual Screening Predicted Hits for Grid B (RKS)

**Grid B**		
Compound	Structure	XP Glide Score(s)
**13**	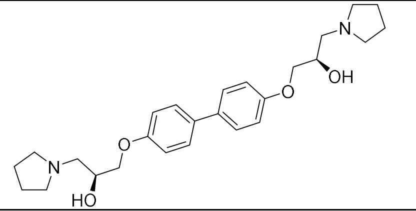	-8.218<br>-7.661<br>-7.206
**14**	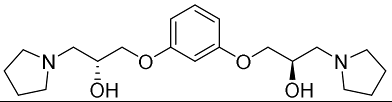	-8.18<br>-8.126<br>-8.058
**15**	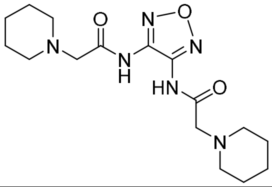	-7.761<br>-7.7017<br>-7.011
**16**	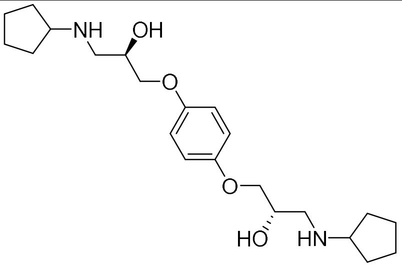	-7.525<br>-7.477<br>-7.453<br>-7.442<br>-7.034
**17**	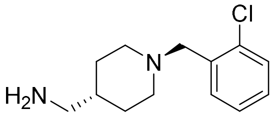	-7.448<br>-7.187
**18**	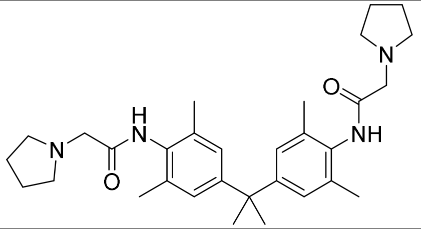	-7.425
**19**	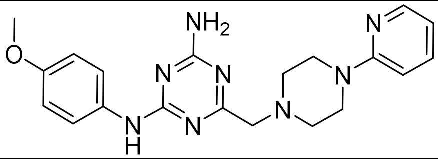	-7.318<br>-7.249<br>-7.129
**20**	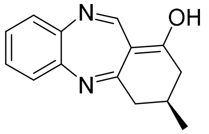	-7.299<br>-7.068
**21**	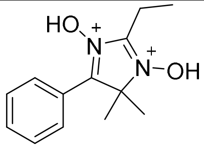	-7.253

**22**	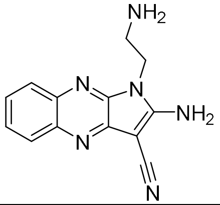	-7.246<br>-7.214
**23**	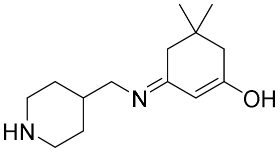	-7.155<br>-7.088
**24**	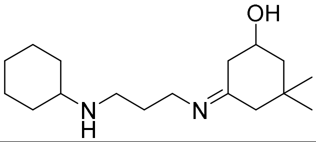	-7.114
**25**	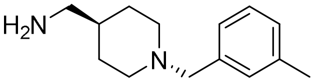	-7.096
**26**	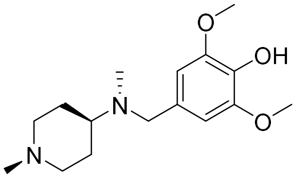	-7.078
